# Patients’ Use of e-Consultations as an Alternative to Other General Practitioner Services: Cross-Sectional Survey Study

**DOI:** 10.2196/55158

**Published:** 2025-01-08

**Authors:** Eli Kristiansen, Helen Atherton, Bjarne Austad, Trine Strand Bergmo, Børge Lønnebakke Norberg, Chris Salisbury, Paolo Zanaboni

**Affiliations:** 1 Department of Clinical Medicine Faculty of Health Sciences UiT The Arctic University of Norway Tromsø Norway; 2 Norwegian Centre for E-health Research University Hospital of North Norway Tromsø Norway; 3 School of Primary Care Population Health and Medical Education University of Southampton Southampton United Kingdom; 4 General Practice Research Unit Department of Public Health and Nursing Norwegian University of Science and Technology Trondheim Norway; 5 Centre for Academic Primary Care, Population Health Sciences Bristol Medical School University of Bristol Bristol United Kingdom

**Keywords:** e-consultation, remote consultation, telehealth, primary care, general practitioner, patient experience, cross-sectional, digital health, survey

## Abstract

**Background:**

e-Consultations, defined as asynchronous text-based messaging, have transformed how patients interact with their general practitioner (GP). While e-consultations can improve patient access to GP care, concerns about increased workload for GPs are raised.

**Objective:**

This study aimed to address three research questions: (1) For what purpose and with what expectations do patients initiate e-consultations? (2) If e-consultations had not been available, what alternative actions would the patient have taken? and (3) How are the alternative actions associated with patient and e-consultation characteristics?

**Methods:**

A cross-sectional study was conducted through a web-based survey on Helsenorge. Helsenorge is the national citizen portal for digital health services in Norway, including e-consultations with the GP. All users who sent e-consultations through Helsenorge were invited to participate between January and February 2023. The survey addressed questions on users’ expectations and experience with e-consultations. The association between patient and e-consultation characteristics and alternative actions to e-consultations were analyzed using multinomial logistic regression.

**Results:**

Overall, 13,011 users answered the survey. The most common reason for initiating an e-consultation was requesting a sick certificate (4940/13,011, 38%). Overall, 68.7% (8802/13,011) of respondents expected an answer within 24 hours, and 17.7% (2310/13,011) anticipated that the GP would ask them to attend a physical examination. If e-consultations had not been available, 45.5% (5917/13,011) of respondents would have booked a GP appointment, and 44.9% (5846/13,011) would have called the front desk. Users who expected a quicker response (odds ratio [OR] 1.64, 95% CI 1.46-1.85) and were less concerned about their health issues (OR 1.29, 95% CI 1.18-1.40) were more likely to call the front desk. Only 2.5% (323/13,011) of respondents would have contacted out-of-hours services. Users with longer travel time to the GP office (OR 6.08, 95% CI 3.46-10.66) and with a new health problem (OR 2.71, 95% CI 2.09-3.51) were more likely to choose this option. In addition, 4.7% (609/13,011) of the users would not have sought help if e-consultations had not been available. Younger patients (OR 2.16, 95% CI 1.38-3.37) and those with a longer travel time to the GP office (OR 2.19, 95% CI 1.27-3.80) or a new health issue (OR 1.74, 95% CI 1.43-2.12) had higher odds for not seeking help.

**Conclusions:**

e-Consultations were often the patients’ first choice of access route, and users expected a fast response. e-Consultations were mostly perceived as an alternative to GP appointments or calling the front desk. Patients with lower availability to the GP office had higher odds of using e-consultations as an alternative to out-of-hours service or waiting and not seeking GP care. Guidance for patient use should be developed to ensure appropriate and safe use. Further research should assess the effect of e-consultations on health outcomes and efficiency.

## Introduction

### Background

The increased availability of e-consultations with general practitioners (GPs) has raised questions about how they affect patients’ health care service use. An e-consultation consists of asynchronous text-based messaging between the patient and GP [1]. Norwegian authorities state that e-consultations must be used to solve clinical problems [2] and expect that e-consultations will be used as an alternative to physical appointments to relieve GPs from some of the workload pressure [3]. Users perceive e-consultations as more accessible and available than physical GP consultations [4-6]. At the same time, GPs have reported increased workload due to patients seeking help for minor issues [5,7] and a need for follow-ups after e-consultations [8].

### e-Consultations in Norwegian General Practice

Remote consultations with the GP can be conducted through e-consultations, video consultations, or telephone consultations. Adoption and use of remote consultations among GPs and patients in Norway experienced significant growth in relation to the COVID-19 pandemic [9]. While e-consultations and telephone consultations were offered by most GPs, video consultations were only used to a limited extent in 2023 [10].

According to Norwegian authorities, e-consultations must provide medical advice, and GPs should generally respond within 5 working days. Patients pay the same out-of-pocket fee for e-consultations as for face-to-face GP appointments [2]. In most situations, e-consultations are initiated by patients. However, there are no official guidelines to assist patients in assessing if their issues can be handled through e-consultations.

### Theories and Knowledge About the Use of and Access to e-Consultations

The implementation of e-consultations has changed patients’ access to GPs and possibly affected the patients’ way of seeking health care [11]. Patients’ adoption and use of e-consultations are affected by demographic characteristics [5,12] and health needs [13,14], in addition to perceived availability, accessibility, and capability to use the service [4,15]. Perceived availability and accessibility of e-consultations can, in turn, depend on factors such as self-selected use, information about the service, patient expectations and experience, expected waiting time for a GP appointment, and cost of service [4,5,16,17].

Research shows that patients value e-consultations for their high accessibility and efficiency [18-21]. e-Consultations are primarily used for less severe health concerns [18] and already-known problems [22]. Patients are less willing to use e-consultations for serious issues [20] and think that complex issues should not be addressed in e-consultations [21]. However, a study from Denmark showed that the complexity of the content of e-consultations was high in terms of both the number of questions asked and the disease perspective [23]. An established relationship between GP and patient facilitates good communication around clinical issues and mutual trust in e-consultations [4,24]. If e-consultations are to reduce the workload for GPs, they should be used as an alternative to a face-to-face consultation without the need for a follow-up examination at the GP office [7,25]. However, there is a lack of clear evidence regarding the circumstances under which an e-consultation can effectively replace a face-to-face consultation and the potential impacts of such use [5]. Some studies show that e-consultations are not used as a replacement for face-to-face consultations but rather as an additional service [5,6,26]. Research shows that patients can use e-consultations as an alternative to calling the front desk (ie, patients asking the GP for help to triage their health issues) [3,27,28]. Another study suggests that the high availability of e-consultations can reduce visits to out-of-hours primary care services [29].

It has been argued that e-consultations should not replace other general practice services, instead, they should be viewed as a complementary service that adds flexibility to general care [4,7,18,30,31]. The flexibility and availability of e-consultations can generate new demand for GP care. By lowering patients’ threshold for contact, e-consultations may lead to requests that would not have otherwise entered the health care system, thereby increasing the GP workload [25,27]. However, it remains unclear whether this new demand arises from previously unmet needs due to limited access or from patients reaching out more readily for minor issues [20,25].

### Study Aim

This study aimed to investigate how users perceived their use of e-consultations. Did they consider it an alternative to other GP services, or was a new demand for GP care created? We defined other GP services as GP appointments, phone calls to the front desk, or out-of-hours services. Information about circumstances for use and how patients perceived the role of e-consultations in the general practice setting is important for future service development.

The research questions (RQs) addressed by this study were as follows: (RQ1) For what purpose and with what expectations do patients initiate e-consultations? (RQ2) If e-consultations had not been available, what alternative actions would the patient have taken? and (RQ3) How are the alternative actions associated with patient and e-consultation characteristics?

## Methods

### Study Setting

General practices in Norway are organized in a scheme where citizens can choose a specific GP. The average patient list per GP was approximately 1040 patients in 2023 [32]. GPs serve as gatekeepers to specialized health care services and provide welfare services such as sick certifications. They have substantial autonomy in organizing their practices, including the option to offer e-consultations. Most GPs in Norway are responsible for their own GP list but share office locations and support personnel with other GPs. On average, the Norwegian citizen conducted 3 consultations (including e-consultations) with the GP in 2023 [33]. In recent years, the GP scheme has had problems with high work pressure [34]. The national health portal, Helsenorge, hosts several digital health services for citizens and is the main platform for digital GP services, including e-consultations. GPs are also responsible for providing out-of-hours services and taking turns staffing clinics outside regular hours. The out-of-hours service addresses urgent health care needs, such as acute illnesses, injuries, or conditions that cannot wait until regular clinic hours. The Norwegian health care system offers free GP care with minimal out-of-pocket expenses for patients. In January 2023, patients paid the same amount for regular consultations and e-consultations, 160 NOK (US $14.36) or 212 NOK (US $19.03) for consultations with GPs without or with specialization in general practice, respectively. In addition to the out-of-pocket fee from patients, GPs receive extra payment per consultation from the reimbursement system. GPs received the same total amount for both regular consultations and e-consultations, 175 NOK (US $15.71) without specialization or 285 NOK (US $25.59) with specialization [2].

### Study Design

We conducted a cross-sectional study through a nationwide web-based survey of e-consultation users. The user survey was developed by the study authors, who have expertise in digital health and primary health care. It was based on academic knowledge and previous research on digital health care use and theories of access to health care [4,12,13,15-17,22]. Textbox 1 shows the survey themes. The survey was in Norwegian. The whole survey, and English translations can be found in Multimedia Appendix 1.

**Overview of survey topics.**

**Patient’s background characteristics**

1. Gender

2. Age

3. Education

4. Health care use: both general practitioner (GP) appointments and e-consultations

5. Digital maturity: first-time user or not

6. Proxy user

**Availability of GP services**

7. Travel time to the GP office

8. Ease of getting through on the phone to the doctor’s front desk

9. Ease of getting a GP appointment within a reasonable time

**Use of and access to e-consultations**

10. Information about the service

11. Used as the first choice for issues in the matter

12. Expected response time

13. Cost of service

14. Satisfaction with the use of e-consultations

15. Alternative action if e-consultations had not been available

**Characteristics of the problem handled in the e-consultation**

16. Level of concern about the problem

17. Perceived suitability of issue to be handled digitally

18. Reason for sending e-consultation

Experts from Norsk Helsenett, the national service provider of the health portal Helsenorge, revised the survey. The survey was piloted on 5 e-consultation users in different age groups. Norsk Helsenett facilitated the survey invitation on the web page Helsenorge.no. All users who sent an e-consultation to their GP through Helsenorge in 3 weeks from Monday, January 30, 2023, to Sunday, February 2, 2023, received an invitation to the survey. The invitation was presented through a pop-up on the web page and read “Do you want to participate in research about e-consultations?” The e-consultation service is only accessible through a secure log-in. If a user sent more than one e-consultation during the study period, a pop-up would appear for each e-consultation they sent. The respondents were informed that participation in the survey was voluntary and anonymous. They gave consent by clicking “I agree to be a part of the survey” at the bottom of the page and were then redirected to an external web page containing the survey. The web-based data collection solution used was Questback Essential.

The survey had up to 20 questions (depending on the respondent’s answers) distributed over 15 pages. The survey was written in Norwegian, and pilot estimations showed that the survey took about 7 minutes to complete. All questions were mandatory to answer. The questions had multiple choice answers, except for 3 questions about availability and use that were answered through a 5-point Likert scale. Only completed questionnaires were analyzed. It was not possible to calculate the completion rate since no information about uncompleted questionnaires was provided. We used the STROBE (Strengthening the Reporting of Observational Studies in Epidemiology) checklist for cross-sectional studies [35] (Multimedia Appendix 2) and the CHERRIES (Checklist for Reporting Results of Internet E-Surveys) [36] when reporting the results (Multimedia Appendix 3).

### Data Analysis

The response rate was calculated based on routine data about all patient-initiated e-consultations sent through Helsenorge during the 3-week period.

RQ1 was answered using descriptive statistics, including the frequency and percentages of all respondents. Survey topics 10-18 (except 15) were analyzed (Textbox 1).

RQ2 was answered through survey topic 15 (“If you had not had the opportunity to send an e-consultation, what would you have done?”). The answer options to this question were book a GP appointment, call the front desk, wait or seek information on the internet, or take other actions. These are hereafter called alternative actions. Descriptive statistics were reported, including the frequency and percentages of all respondents.

RQ3 was examined through a multinomial logistic regression. Conducting a regression made it possible to investigate the association between the characteristics of the patients and e-consultations and their alternative action to sending the e-consultation. The dependent variable was the users’ assessment of alternative actions to using e-consultation (survey topic 15). The reference group of the analysis was the users choosing “booking a GP appointment.” This group was chosen since e-consultations aim to be an alternative to physical GP appointments for their most effective use. We compared it with users who would rather have called the front desk, contacted out-of-hours service, or waited or searched for information on the internet. This gave reasonable indications of use patterns and reasons for sending an e-consultation in different circumstances. The group that answered “other actions” was not included in the analysis. The respondents of this group are presumably heterogeneous, making it difficult to analyze their group characteristics meaningfully. We included all other survey topics as independent variables in the regression, except the time and day for answering the survey (Textbox 1). For simplicity, the survey questions with 5 answer options were merged into 3 options (“strongly agree” and “agree” were merged to “agree,” “strongly disagree” and “disagree” were merged to “disagree,” “very satisfied” and “satisfied” were merged to “satisfied,” and “very dissatisfied” and “dissatisfied” were merged to “dissatisfied”).

We examined the dataset for multicollinearity before performing the regression, and no indications of multicollinearity were found. As the goal was to investigate associations rather than build a model, we did not present model fit values. The likelihood-ratio test showed that not all variables were statistically significant; however, we included them all to not bias the results. We presented the odds ratio (OR) and 95% CI. The *P* value for statistical significance was set to .05. All analyses were conducted using SPSS (version 25; IBM Corp).

### Ethical Considerations

The study and the data handling procedure were approved by the Data Protection Officer of the University Hospital of North Norway (#03057). According to the Norwegian Act on Medical and Health Research §2 and §4, the study did not require approval from the ethics committee. The study involved human participants. Participants were informed that they gave their consent to participate in the study by answering the survey, and no compensation to participants was given. All data were anonymous.

## Results

### Respondent Statistics

During the period the survey was available, 163,977 patient-initiated e-consultations were sent in the portal. A total of 13,648 e-consultations were assessed by users in the survey, giving a response rate of 8.32%. After two and a half days, we were informed that the question “If you had not had the opportunity to send an e-consultation, what would you have done?” was mistakenly set up as a multiple-choice question. This was corrected to a single-choice format. Respondents who had provided multiple answers (n=637) were removed, leaving a final sample of 13,011 responses.

Table 1 lists the characteristics of the respondents. A total of 78.1% (10,155/13,011) of the respondents had less than 30 minutes of travel time to get to the GP office. Almost half of the respondents (6095/13,011, 46.8%) agreed that it was usually easy to get through on the phone to the front desk, and 64.3% (8366/13,011) agreed that they usually got a GP appointment within a reasonable time.

**Table 1:** Characteristics of survey respondents (N=13,011).

Topic and options					Value, n (%)
**Gender**					
	Female			9059 (69.6)	
	Male			3848 (29.6)	
	Other or I don’t want to answer			104 (0.8)	
**Age range (years)**					
	16-25			823 (6.4)	
	26-40			3595 (27.6)	
	41-55			4764 (36.6)	
	56-70			3123 (24)	
	≥71			706 (5.4)	
**Highest completed education level**					
	10 years of primary school or less			795 (6.1)	
	Upper secondary school			2753 (21.2)	
	Vocational school			2170 (16.7)	
	University less than 4 years			3156 (24.2)	
	University more than 4 years			3875 (29.8)	
	Other			262 (2)	
**Number of GP^a^ appointments in the last 12 months**					
	0-3			6236 (47.9)	
	4 -9			5572 (42.8)	
	10-19			1029 (7.9)	
	20 or more			174 (1.4)	
**Number of e-consultations in the last 12 months**					
	1-3			6646 (51.1)	
	4-9			4607 (35.4)	
	10-19			1358 (10.4)	
	20 or more			400 (3.1)	
**Proxy user?**					
	No, sending it on behalf of myself			11,828 (90.9)	
	Yes, on behalf of my child			1068 (8.2)	
	Yes, on behalf of others I have power of attorney for			115 (0.9)	
**Time of answering the survey**					
	06:00-09:59 (day)			3199 (24.6)	
	10:00-13:59 (day)			4532 (34.8)	
	14:00-17:59 (day)			2483 (19.1)	
	18:00-21:59 (day)			1825 (14)	
	22:00-01:59 (night)			803 (6.2)	
	02:00-05:59 (night)			169 (1.3)	
**Day of answering the survey**					
	Monday			3091 (23.8)	
	Tuesday			2245 (17.3)	
	Wednesday			2338 (18)	
	Thursday			2364 (18.2)	
	Friday			1660 (12.8)	
	Saturday			371 (2.9)	
	Sunday			942 (7)	
**User’s perception of the availability of GP services**					
	**Travel time to GP office**				
		0-30 minutes	10,155 (78.1)		
		30-60 minutes	2178 (16.7)		
		1-2 hours	468 (3.6)		
		More than 2 hours	210 (1.6)		
	**It is usually easy to get through on the phone to my GP office front desk**				
		Strongly agree	2199 (16.9)		
		Agree	3896 (29.9)		
		Neither agree nor disagree	3365 (25.9)		
		Disagree	2272 (17.5)		
		Strongly disagree	1279 (9.8)		
	**I usually get a GP appointment within a reasonable time**				
		Strongly agree	3601 (27.7)		
		Agree	4765 (36.6)		
		Neither agree nor disagree	2513 (19.3)		
		Disagree	1489 (11.5)		
		Strongly disagree	643 (4.9)		

^a^GP: general practitioner.

### Use of e-Consultations: for What Reasons and With What Expectations Were e-Consultations Initiated?

Most respondents (10,351/13,011, 79.7%) used e-consultations as their first choice to contact the GP that day, and 83.9% (10,913/13,011) were satisfied with using it.

The results indicate a lack of information about the service given to users. More than half (7466/13,011, 57.4%) of the users had not received information about the possibility of sending an e-consultation but found out about the service by themselves. As many as 20.5% (2670/13,011) of the respondents did not know whether or not they had to pay to send an e-consultation. Recommendations for GPs’ use of e-consultations state that an answer should be given within 5 days. However, more than two-thirds (8802/13,011, 68.7%) of the respondents expected a response to their e-consultations within 24 hours.

Only a minority of the e-consultations (1824/13,011, 14%) dealt with problems the user was very worried about. A total of 17.8% (2310/13,011) thought that the e-consultation would result in the GP asking them to come to the office for a physical examination (Table 2).

Table 3 shows the reasons why a patient sent an e-consultation. Multiple answers were permitted. Most e-consultations contained a request for a sick certification (4940/13,011, 38%) or were about a known problem (4778/13,011, 36.7%). A new health problem was the reason for 23.2% (3015/13,011) of the consultations. Only 8.5% (1110/13,011) of the e-consultations were initiated to ask the GP whether they should book a GP appointment for their problem.

**Table 2:** Patient’s perception of access and use of e-consultations (N=13,011).

Topic and options				Value, n (%)
**Access and use of e-consultations**				
	**Who told you that sending an e-consultation to the GP^a^ is possible?**			
		Found the service myself	7466 (57.4)	
		The GP or the health receptionist	4553 (35)	
		Brochures, advertisements, or other written material	101 (0.8)	
		Friends, family, or colleagues	652 (5)	
		Other	239 (1.8)	
	**Sending an e-consultation was my first choice to get an answer from my GP about my problem today.**			
		Strongly agree	7497 (57.6)	
		Agree	2872 (22.1)	
		Neither agree nor disagree	1410 (10.8)	
		Disagree	804 (6.2)	
		Strongly disagree	428 (3.3)	
	**Expected time to get an answer to this e-consultation**			
		Within 12 hours	4445 (34.2)	
		Within 24 hours	4356 (33.5)	
		Within 48 hours	2340 (18)	
		Between 48 hours and 5 days	1860 (14.3)	
	**Getting the e-consultation for free?^b^**			
		Yes	1140 (8.8)	
		No	9201 (70.7)	
		Don’t know	2670 (20.5)	
	**Would you have sent this e-consultation if you had to pay for it?^c^ (n=1150)**			
		Yes	870 (76.3)	
		No	141 (12.4)	
		I don’t know	139 (11.3)	
**Characteristics of the problem handled in the e-consultation**				
	**How concerned are you about the issue you sent an e-consultation for?**			
		Not worried	4744 (36.5)	
		Somewhat worried	6443 (49.5)	
		Very worried	1824 (14)	
	**Do you think the GP will answer this e-consultation by asking you to come to the office for a physical examination?**			
		Yes	2310 (17.7)	
		No	5396 (41.5)	
		Don’t know	5305 (40.8)	
	**All in all, how satisfied were you with contacting the GP through an e-consultation today?**			
		Very satisfied	6495 (49.9)	
		Satisfied	4418 (34)	
		Neither satisfied nor dissatisfied	1776 (13.6)	
		Dissatisfied	221 (1.7)	
		Very dissatisfied	101 (0.8)	

^a^GP: general practitioner.

^b^Some patient groups receive free health care services, including e-consultations (eg, pregnant women, first-time military service members, and patients with high costs of health care services receiving exemption cards).

^c^Question was only asked to the ones who answered that they got the e-consultation for free.

**Table 3:** Patients’ reasons for sending an e-consultation (N=13,011).

Reasons	Value, n (%)
Sick certificate or other certificates	4940 (38)
Known health problems	4778 (36.7)
New health problems	3015 (23.2)
Medication use	1595 (12.3)
Other issues	1537 (11.8)
Asked if I needed to book a GP^a^ appointment	1110 (8.5)
Test results	943 (7.2)
Answered an e-consultation from the GP	453 (3.5)

^a^GP: general practitioner.

### Handling of the Medical Problem if e-Consultations had not Been Available

Nearly 9 out of 10 respondents would have contacted the GP office if the e-consultation service had not been available. Only 4.7 % (609/13,011) of the patients would not have contacted health care services but rather sought information on the internet or waited Table 4).

**Table 4:** Patients’ assessment of alternative action if the e-consultation service had not been available (N=13,011).

Alternative action	Value, n (%)
Book a GP^a^ appointment	5917 (45.5)
Call the front desk	5846 (44.9)
Contact out-of-hours service	323 (2.5)
Wait or seek information on the internet	609 (4.7)
Other	316 (2.4)

^a^GP: general practitioner.

### Factors Associated With Using e-Consultation as an Alternative to Other Actions

#### Overview

Table 5 shows the results from the multinomial regression model. The table presents associations between the characteristics of patients and e-consultations and how the respondent would have handled the medical problem if e-consultations had not been available. The reference category is “Book a GP appointment.” Associations with the patient’s alternative options (calling the front desk, contacting out-of-hours, and waiting or seeking information) relative to booking a GP appointment are reported with all other variables held constant. Descriptive statistics of all groups are found in Multimedia Appendix 4.

**Table 5:** Associations between characteristics of patients and e-consultations and their perception of what they alternatively have done if e-consultations had not been available.

					Call front desk (n=5846)		Out-of-hours service (n=323)		Wait or seek information (n=609)^a^
					OR^b^ (95% CI)		OR (95% CI)		OR (95% CI)
**Background patient characteristics**									
	**Gender (reference: female)**								
		Male		0.85*** (0.78-0.93)		1.26 (0.98-1.63)		0.94 (0.77-1.14)	
	**Age range (years; reference: ≥71)**								
		16-25		1.15 (0.91-1.45)		1.44 (0.74-2.81)		2.16*** (1.38-3.37)	
		26-40		1.03 (0.85-1.24)		1.07 (0.61-1.85)		1.29 (0.89-1.86)	
		41-55		0.91 (0.76-1.08)		0.88 (0.52-1.51)		0.75 (0.52-1.07)	
		56-70		0.96 (0.80-1.16)		0.90 (0.52-1.56)		0.69* (0.47-1.0)	
	**Education (reference: university education)^c^**								
		Nonuniversity education		1.12* (1.0-41.22)		1.24 (0.98-1.58)		0.99 (0.83-1.19)	
	**Number of GP^d^ appointments in the last 12 months (reference: 1-3)**								
		4-9		1.07 (0.98-1.16)		1.13 (0.87-1.47)		1.02 (0.84-1.24)	
		10-19		1.05 (0.90-1.22)		1.31 (0.84-2.05)		1.07 (.077-1.50)	
		20 or more		1.09 (0.77-1.55)		2.75* (1.29-5.86)		1.24 (0.59-2.59)	
	**Number of e-consultations in the last 12 months (reference: 1-3)**								
		4-9		1.06 (0.97-1.16)		1.04 (0.79-1.38)		1.01 (0.82-1.24)	
		10-19		1.26** (1.10-1.44)		1.33 (0.88-2.02)		1.19 (0.87-1.62)	
		20 or more		1.30* (1.02-1.65)		1.01 (0.49-2.07)		1.45 (0.89-2.38)	
	**First time sending e-consultation (reference: no)^e^**								
		Yes		1.00 (0.88-1.13)		0.88 (0.61-1.27)		0.90 (0.68-1.18)	
**Availability of GP services**									
	**Travel time to GP office (reference: less than 30 minutes)**								
			30-60 minutes	0.97 (0.87-1.07)		1.55* (1.17-2.05)		1.01 (0.80-1.27)	
			1-2 hours	1.05 (0.86-1.29)		2.22** (1.36-3.61)		1.48 (0.99-2.22)	
			>2 hours	1.27 (0.92-1.76)		6.08*** (3.46-10.66)		2.19** (1.27-3.79)	
	**I usually get through on the phone to my doctor’s front desk (reference: neither agree nor disagree)**								
		Agree		1.07 (0.98-1.18)		0.99 (0.73-1.34)		0.84 (0.68-1.03)	
		Disagree		1.03 (0.92-1.14)		1.11 (0.82-1.51)		0.96 (0.76-1.21)	
	**I usually get a GP appointment within a reasonable time (reference: neither agree nor disagree)**								
		Agree		1.03 (0.93-1.14)		0.58*** (0.43-0.77)		1.04 (0.83-1.31)	
		Disagree		0.96 (0.85-1.09)		0.91 (0.65-1.27)		1.08 (0.81-1.43)	
**Use of and access to e-consultations**									
	**Received information about e-consultations (reference: info from HCP^f^)^g^**								
		No info from HCP		1.11 (1.03-1.20)		0.92 (0.72-1.17)		1.46*** (1.20-1.78)	
	**Sending an e-consultation was my first choice (reference: neither agree nor disagree)**								
		Agree		0.89 (0.78-1.01)		1.19 (0.81-1.74)		1.15 (0.85-1.54)	
		Disagree		1.07 (0.90-1.26)		1.45 (0.91-2.30)		0.81 (0.52-1.25)	
	**Expected response time (reference: between 48 hours and 5 days)**								
		Within 12 hours		1.64*** (1.46-1.85)		2.10*** (1.43-3.08)		0.84 (0.65-1.08)	
		Within 24 hours		1.29*** (1.14-1.45)		1.35 (0.91-2.01)		0.77* (0.60-0.99)	
		Within 48 hours		1.03 (0.90-1.17)		0.66 (0.40-1.09)		0.70** (0.53-0.92)	
	**Getting e-consultation for free (reference: don’t know)**								
		Yes		0.72*** (0.62-0.84)		0.67 (0.40-1.11)		0.73 (0.51-1.04)	
		No		0.71*** (0.64-0.78)		0.84 (0.62-1.12)		0.73* (0.59-0.90)	
**Characteristics of the problem handled in e-consultation**									
	**How concerned were you about the issue? (reference: somewhat worried)**								
		Not worried		1.29*** (1.18-1.40)		0.76 (0.55-1.05)		1.28* (1.06-1.55)	
		Very worried		1.02 (0.90-1.14)		2.71*** (2.07-3.54)		0.88 (0.67-1.1)	
	**Do you think the GP will ask for a physical examination? (reference: don’t know)**								
		Yes		1.02 (0.92-1.14)		1.06 (0.80-1.41)		0.76* (0.60-0.98)	
		No		1.37*** (1.26-1.49)		0.70* (0.52-0.95)		1.05 (0.86-1.29)	
	**Reason for sending e-consultations**								
		Sick certification or other certification		1.07 (0.98-1.16)		0.94 (0.72-1.25)		0.31*** (0.24-0.39)	
		New problem		0.94 (0.85-1.03)		2.71*** (2.09-3.51)		1.74*** (1.43-2.12)	
		Former known problem		0.62*** (0.57-0.68)		0.71* (0.54-0.92)		0.69*** (0.57-0.84)	
		Medicine use		1.04 (0.92-1.17)		0.96 (0.65-1.40)		1.16 (0.91-1.48)	
		Test results		1.31*** (1.13-1.52)		0.53* (0.29-0.97)		1.27 (0.93-1.74)	
		Ask if needed to order an appointment		0.97 (0.84-1.11)		1.13 (0.79-1.63)		0.90 (0.67-1.22)	
		Answer question from GP		1.11 (0.90-1.38)		1.20 (0.64-2.25)		1.54* (1.01-2.33)	
	**Proxy user (reference: no)**								
		Yes		1.26** (1.09-1.45)		1.59* (1.06-2.37)		0.60* (0.42-0.84)	
**Satisfaction with e-consultations**									
	**How satisfied were you with sending the e-consultation today? (reference: neither satisfied nor dissatisfied)**								
		Satisfied		0.74*** (0.66-0.83)		0.49*** (0.36-0.66)		0.79 (0.60-1.02)	
		Not satisfied		1.22 (0.92-1.61)		0.84 (0.44-1.61)		1.39 (0.77-2.52)	

^a^The dependent variable “wait or seek information” consists of two options: “Sought information on the internet for my problem” and “waited a while.”

^b^OR: odds ratio.

^c^Variable about education was merged into two categories: with or without university education.

^d^GP: general practitioner.

^e^We constructed a dummy variable for first-time users based on the question “Are you a first-time user?”

^f^HCP: health care personnel.

^g^Variable about receiving information about service was merged into two categories: “Info from HCP” and “No info from HCP” including “found the service by myself,” “brochures, advertisements/other material,” “friends/family/colleagues,” and “other”

*P<.05, **P<.01*,* ***P<.001

#### Calling the Front Desk Relative to Booking a GP Appointment

The patients who expected a response within 12 hours (OR 1.64, 95% CI 1.46-1.85) and 24 hours (OR 1.29, 95% CI 1.14-1.45) had higher odds of calling the front desk compared with those who expected to wait 5 days for an answer.

The respondents who were not worried about the issue handled in the e-consultation had higher odds (OR 1.29, 95% CI 1.18-1.40) of calling the front desk compared with those who were somewhat worried.

The patients who thought the GP would not ask them to come in for a physical examination had higher odds of contacting the front desk (OR 1.37, 95% CI 1.26-1.49) compared with the group who said they did not know if the GP would recommend a physical examination.

The patients who asked about test results had higher odds (OR 1.31, 95% CI 1.13-1.52) of contacting the front desk in comparison to the ones who did not use e-consultations to ask about test results.

#### Contacting the Out-of-Hours Service Relative to Booking a GP Appointment

Among the respondents who had more than 30 minutes of travel time to the GP office, the odds of contacting out-of-hours service were much higher (30-60 minutes: OR 1.55, 95% CI 1.17-2.05; 1-2 hours: OR 2.22, 95% CI 1.36-3.61; over 2 hours: OR 6.08, 95% CI 3.46-10.66) compared with those who had less than 30 minutes of travel time.

The odds for contacting out-of-hours services were higher (OR 2.10, 95% CI 1.43-3.08) for those who expected an answer within 12 hours compared with those who expected an answer within 5 days.

For patients who usually get a GP appointment within a reasonable time, the odds of contacting out-of-hours service were lower (OR 0.58, 95% CI 0.43-0.77) compared with patients who answered that they thought GP appointments were neither available nor unavailable.

Among the patients who were very worried about their health issues, the odds of contacting out-of-hours service were higher (OR 2.71, 95% CI 2.07-3.54) compared with those who were only somewhat worried about their health issues.

For patients who had a new problem, the odds of contacting out-of-hours service were higher (OR 2.71, 95% CI 2.09-3.51) compared with those who did not have a new problem.

#### Waiting or Seeking Information on the Internet Relative to Booking a GP Appointment

Among the patients aged 16-25 years, the odds for waiting or seeking information on the internet were higher (OR 2.16, 95% CI 1.38-3.37) than for users over 70 years old.

Also, among patients with a particularly long travel time to the GP office (more than 2 hours), the odds for waiting or seeking information on the internet were higher (OR 2.19, 95% CI 1.27-3.80) than users with a shorter travel time.

The respondents who expected a short response rate had lower odds (OR 0.77, 95% CI 0.60-0.99 and OR 0.70, 95% CI 0.53-0.92) for choosing the waiting or seeking information on the internet alternative compared with those expecting a response rate of over 48 hours.

Among the respondents who had not received information about the service from health care personnel, the odds of waiting or seeking information on the internet were higher (OR 1.15, 95% CI 1.20-1.78) than the ones who had received information.

If the patients’ reason for sending an e-consultation was a new problem, the odds of choosing to wait or seek information on the internet were higher (OR 1.74, 95% CI 1.43-2.12) compared with users who did not ask about a new problem.

## Discussion

### Principal Findings

This web-based survey gathered experiences from 13,011 users of e-consultation in Norway. Most had short distances to travel to the GP office, but still, 79.6% (10,369/13,011) reported that e-consultations were their first choice for contacting the GP. The 2 most common reasons for sending an e-consultation were obtaining a sick certification or asking a question about an already known health problem. In addition, 1 out of 4 e-consultations concerned a new health issue. However, only 14% (1824/13,011) of the requests addressed issues the patient was worried about. Almost 70% (8801/13,011, 67.7%) of the users expected to receive an answer from their GP within 24 hours, despite the Norwegian recommendation to GPs to provide an answer within 5 working days.

If e-consultations had not been available, 45.5% (5917/13,011) of the patients would have booked a physical GP appointment, and 44.9% (5846/13,011) would have contacted the front desk. Patients who alternatively would have called the front desk were associated with not worrying about their health problems and not thinking a physical examination would be necessary. Few patients (323/13,011, 2.5%) answered that they would alternatively have contacted out-of-hours services. These were associated with longer travel distances to the GP office and low availability of GP appointments. Finally, 4.7% (609/13,011) responded that waiting or seeking information on the internet was their potential alternative for sending an e-consultation. These were associated with younger age, a new health problem they did not worry about, and long travel distances to the GP office.

### Strengths and Limitations

Despite a low response rate, which is often the case in web-based surveys, the web-based survey design facilitated an efficient collection of a large sample size. Reminders may have increased the response rate. However, as the survey was made available through a pop-up in the web browser and no e-mail addresses or other identifiers were collected, there was no possibility of sending survey reminders. Since the pop-up with the invitation to the survey appeared for each e-consultation sent, it was potentially possible for a user to answer more than once.

The Norwegian official register data for remote consultations do not distinguish between e-consultations, video consultations, and telephone consultations [9]. No specific data on users of e-consultations on Helsenorge are available. However, Norsk Helsenett have provided us with the total number of e-consultations sent in the portal during the survey period. Because no data are available on the characteristics of e-consultation users, it was not possible to assess the overall representativeness of the survey respondents. We could only rely on the large number of respondents.

The respondents were invited to answer the survey immediately after sending an e-consultation. Therefore, the patients’ answers were not biased by the GP’s response to the e-consultation. Since data were collected after the COVID-19 pandemic, we can assume that e-consultations were used for all purposes and not mainly to minimize the spread of infections.

We recognize that different appointment booking systems among general practices could lead to inconsistency in answers about alternative actions for e-consultation. Most general practices have a web-based booking system, but some still arrange appointment bookings by telephone at the front desk. In addition, practices can operate with on-the-day acute appointments only available by booking from the front desk. This could have made it hard for some respondents to choose whether e-consultation was an alternative to calling the front desk or booking a GP appointment. The differences we found between the groups should be interpreted with care.

The regression analysis was based on the simplified assumption that e-consultations were used as an alternative or replacement for other GP services. In real life, a patient may intentionally want to use e-consultations for their request without reflecting if it replaces another service. In this setting, e-consultations have their own valuable and independent role in helping patients manage their health. Despite this simplification of actual use, our analysis provides insights into the circumstances of using e-consultations in different settings. It also provides knowledge of patients’ perception of using e-consultations compared with the other GP services.

The study did not measure the outcome of the e-consultation (eg, how many e-consultations required an immediate follow-up at the GP office or how many were perceived as solved in writing). Therefore, we cannot conclude whether the e-consultation service adds extra workload to the GP due to the need for follow-up after e-consultations.

### Comparison With Previous Work

#### Use of e-Consultations

This study confirms previous findings demonstrating that users are satisfied with e-consultations and appreciate their high availability [18,31,37]. The expectation of a rapid response time and that most users found out about the service by themselves, probably while navigating the web-based health portal Helsenorge, aligns with findings from a previous study involving older users of e-consultations in Norway [4]. Our study confirms that e-consultations are mostly used for existing medical problems [22]. However, every fourth e-consultation was about a new problem. This is a high share, taking into account that previous research has shown that e-consultations may be less suitable for newly emerged problems [37]. Using e-consultations for sick certificates has been perceived as more efficient and suitable for known health problems [28,38].

#### e-Consultation as an Alternative to Physical Consultations

In 45.5% (5917/13,011) of the assessed e-consultations, the respondent answered that a GP appointment would have been the alternative action for help. For both the patient and the GP, a medical issue solved by sending an e-consultation is convenient and efficient. However, the potential efficiency gain decreases if a physical examination or many follow-up e-consultations are required after the e-consultation [4,23,25]. Almost 20% (2310/13,011, 17.7%) of the patients in our study believed that the GP would answer the e-consultation by asking them to come to the office for a physical examination. This indicates a potential follow-up through a face-to-face examination. Research has shown ambiguous findings related to follow-up contact after digital contact [25]. These findings range from 34% of all e-visits in the United States to 66% of all e-consultations in the United Kingdom needing follow-up after initial digital contact [39,40]. Another study indicates that digital contacts do not lead to more or sooner follow-up consultations than in-person service [41]. Our study did not measure how often an issue was solved in an e-consultation or how often a follow-up was required. Thus, it is not possible to draw a definitive conclusion regarding the impact of e-consultations on efficiency in the Norwegian context.

#### e-Consultations as an Alternative to Calling the Front Desk

Previous research has shown that patients use e-consultations to obtain quick answers to minor issues [4,18,20]. Our study found that 44.9% (5846/13,011) of the users would have contacted the front desk if e-consultations had not been available. These users were associated with health concerns they did not worry about or deem necessary for a physical examination. In addition, the patients who had sent many e-consultations in the last 12 months had higher odds of choosing this alternative. We interpret this as e-consultations that meet a need for communication about minor issues when in-person GP appointments are not necessary. Even for minor issues, these patients had high expectations of a fast response from the GP.

Studies of the eConsult service in England show that issues previously handled by the front desk are directly sent to the GP’s inbox after implementing the service [26,28]. The eConsult service differs from the Norwegian e-consultations service, as it is a web-based triage platform meant to be the first point of contact for triaging and often requires a follow-up [42]. In Norway, front desk personnel usually triage incoming phone calls and advise patients by giving in-person advice and clarifying requests. We believe some of the e-consultations to the GP could have been efficiently handled by the front desk personnel, thus reducing the inbox for the GP.

#### e-Consultations as an Alternative to Out-of-Hours Service

Previous studies have shown that patients seek out-of-hours services when GP offices are closed or are perceived as unavailable [42-44]. The users who answered that out-of-hours services would have been the alternative if e-consultations had not been available were associated with longer travel distances to their GPs. Previous research has shown that the high availability of e-consultations can slightly reduce the demand for out-of-hours services [29]. It raises a concern that users with new and worrisome problems who expect a fast response may turn to e-consultations instead of seeking immediate care. The e-consultation service is unsuitable for urgent care as the response time norm is 5 working days. Ensuring that e-consultations do not become an alternative for urgent care is crucial, as this risks patient safety. The risk of patients using e-consultations for urgent issues has been highlighted as an unintended consequence of e-consultations in another study from England [28].

#### e-Consultations as an Alternative for Waiting or Seeking Information on the Internet

Our study found that 4.7% (609/13,011) of the users who initiated e-consultations would alternatively have waited to seek GP care. Earlier research has also described how e-consultations can create an extra demand for GP care [25,45]. The group that alternatively would have waited or sought information on the internet had higher odds of being younger and addressing a new issue they were less worried about. Previous research supports that better access to primary care prompts seeking help for minor complaints [46]. When e-consultations lower the threshold for use and lead to requests that could just as well be handled outside health care services, the GPs’ health care resources may be misallocated. It can also add to the GP’s workload. On the other hand, our study indicates that users of e-consultations with a long way to the GP office had higher odds of waiting and seeking GP care if e-consultations had not been available. This indicates that e-consultations improve access to GP care for patients who would alternatively not have sought help due to unavailability.

### Implications and Future Research

Users of e-consultations are mainly satisfied, probably due to the increased convenience of e-consultations. Our study indicates a potential efficiency gain for patients and GPs as many patients initiate e-consultations in circumstances where they alternatively would have booked a GP appointment. However, we do not know how many e-consultations would need a follow-up, such as a physical appointment or requesting more information in writing. In addition, our findings suggest a slight increase in demand for GP care and a shift in tasks from front desk staff to GPs, which challenges the total efficiency gain for the GP. The pressure on GPs from patients expecting a fast response can also add to the GPs’ workload. Our study was not designed to investigate efficiency gains or a potential increase or decrease in GP workload, and this question needs further investigation.

Our findings indicate that some of the initiated e-consultations were probably less suitable for the written modality. Examples include e-consultations in which patients deemed a physical examination necessary or where the issue was new or caused the patient significant concern. Knowing that few users had received information about the service from health care personnel and that several were uncertain about the cost of service shows that more information about the service is necessary. Guidance for patients could help ensure appropriate use. This is important for patient safety, keeping GPs’ workload down, and helping GPs prioritize the patients with the most need. Keeping the e-consultation manageable for the GP is important to maintain the offer for the patients. As the service is voluntary for GPs to offer, an overwhelming and unmanageable inbox may drive some GPs to stop providing the service.

Future research is needed to assess the overall efficacy, safety, and impact on GP resource allocation. It should evaluate the added value of e-consultations on health outcomes. A focus should be put on evaluating the added outcome of e-consultation when patients experience increased demand for GP care due to higher availability through e-consultations.

### Conclusion

This study demonstrated that e-consultations are primarily used for convenience, as most respondents reported having good access to their GP due to short travel distances to the GP office and relatively accessible GP appointments. Patients’ expectations for a fast response to the e-consultation were high. Given that the GP norm is a 5-day response time, the proportion of e-consultations addressing new issues and issues the patient was worried about should raise concerns about patient safety. Nearly all patients would have booked a GP appointment or contacted the front desk had e-consultations not been available. This indicates that e-consultations may serve as an alternative to both these parts of the GP service. The study suggests a small increase in demand for GP care through e-consultations for patients who would not have sought health care if e-consultations had not been available. For patients with low availability to the GP office, some e-consultations seemed to be used as an alternative to out-of-hours service or not seeking help. Patients’ concerns about their medical problems influenced how they assessed alternative actions to e-consultations. Those highly concerned had higher odds of alternatively choosing out-of-hours service, while those less concerned were more likely to call the front desk or not seek help. Ensuring appropriate use of e-consultations is crucial for patient safety and managing GP workload. Clear guidance could help patients determine when e-consultations are appropriate. Future research should explore the clinical outcomes and efficiency gains of e-consultations.

## Appendix

Multimedia Appendix 1 Survey questionnaire.

Multimedia Appendix 2 STROBE (Strengthening the Reporting of Observational Studies in Epidemiology) checklist.

Multimedia Appendix 3 CHERRIES (Checklist for Reporting Results of Internet E-Surveys) checklist.

Multimedia Appendix 4 Descriptive statistics cross-table.

## Abbreviations

CHERRIES: Checklist for Reporting Results of Internet E-Surveys
GP: general practitioner
OR: odds ratio
RQ: research question
STROBE: Strengthening the Reporting of Observational Studies in Epidemiology

## Data Availability

The dataset analyzed during this study are available from the corresponding author on reasonable request.
