# Supplementary material for: Patients’ Use of e-Consultations as an Alternative to Other General Practitioner Services: Cross-Sectional Survey Study
Source: J Med Internet Res. 2025 Jan 8;27:e55158. doi: 10.2196/55158 (PMC11754976; doi:10.2196/55158)
Supplement: Multimedia Appendix 1 [file jmir_v27i1e55158_app1.pdf]

Patient survey to users of e-consultations with the GP

Translated from Norwegian.

Presented through a pop-up at Helsenorge.no

1) Who did you send this e-consultation on behalf of?
    a. Your self
    b. Your child
    c. Others you power of attorney for

2) Did you get this e-consultation for free because you have an exemption card, are pregnant, are in military service, or have other conditions that give you free health care?
    a. Yes
    b. No
    c. Don't know

3) *If question 2 is Yes: Would you send this e-consultation if you had to pay for it?*
    a. *Yes*
    b. *No*
    c. *Don't know*

4) Who told you sending an e-consultation to the GP is possible?
    a. The GP or health receptionist
    b. Brochures, advertisements or other written information about e-consultations
    c. Friends, family or colleagues
    d. Figured it out myself, e.j. by searching around in Helsenorge or at the homepage of my GP practice
    e. Other

5) What was the e-consultation about? (more answers are possible)
    a. I asked if I needed to book a GP appointment
    b. Question about a new health problem
    c. Question about my health that I have discussed with my GP earlier
    d. My medication use
    e. Sick certification or other certifications
    f. Test results
    g. Answered a question from the GP
    h. Other issues

6) How worried are you about the problem you sent an e-consultation about?
    a. Not worried
    b. Somewhat worried

c. Very worried

7) If you had not had the opportunity to send an e-consultation, what would you have done?
   a. Booked a GP appointment
   b. Called the front desk
   c. Contacted out-of-hours service
   d. Seek information online or in other places about my health problem
   e. Waited some time
   f. Other actions

8) Do you think the GP will answer this e-consultation by asking you to come to the office for a physical examination?
   a. Yes
   b. No
   c. Don’t know

9) When do you expect an answer to this e-consultation?
   a. Within 12 hours
   b. Within 24 hours
   c. Within 48 hours
   d. Between 48 hours and five days

10) How do you agree or disagree with the following statements?
    (Totally agree, agree, nor agree or disagree, disagree, totally disagree)
    a. Sending an e-consultation was my first choice to get an answer from my GP about my problem today
    b. It is normally easy to get through to my GP office front desk on the phone
    c. I normally get a GP appointment within a reasonable time

11) All in all, how satisfied were you with contacting your GP through an e-consultation today?
    a. Very satisfied
    b. Satisfied
    c. Nor satisfied or dissatisfied
    d. Dissatisfied
    e. Very dissatisfied

12) Do you have any suggestions for improving the e-consultation service? (free text answer)

13) How many e-consultations have you sent to the GP in the last 12 months, including this one?
    a. 1-3
    b. 4-9
    c. 10-19
    d. 20 or more

14) If the answer to question 13 was 1-3: Was this your first e-consultation ever?
    a. Yes

b. *No*

15) How many GP appointments at the GP office have you had in the last 12 months?
    a. 0-3
    b. 4-9
    c. 10-19
    d. 20 or more

16) How long does it usually take you to get to the GP office (travel time from door to door)?
    a. 0-30 minutes
    b. 30-60 minutes
    c. 1-2 hours
    d. More than 2 hours

Finally, some background questions about you:

17) What gender are you?
    a. Female
    b. Male
    c. Other/Don't want to answer

18) How old are you?
    a. 16-25 years
    b. 26-40 years
    c. 41-55 years
    d. 56—70 years
    e. 71-85 years
    f. Over 85 years

19) What is your highest completed education?
    a. 10 years primary school or less
    b. Upper secondary school
    c. Vocational school
    d. University less than 4 years
    e. University more than 4 years
    f. Other

Bruk av e-konsultasjon med fastlegen

Original survey in Norwegian

1) Hvem sendte du denne e-konsultasjon på vegne av?

a. Deg selv
b. Barnet ditt
c. Andre du er pårørende for

2) Fikk du denne e-konsultasjonen gratis fordi du enten har frikort, er gravid, er i førstegangstjeneste eller annet som gir gratis legehjelp?

a. Ja
b. Nei
c. Vet ikke

3) Hvis ja på spørsmål 2: Ville du sendt denne e-konsultasjonen hvis du matte betalt for den?

a. Ja
b. Nei
c. Vet ikke

4) Hvem fortalte deg at det er mulig å sende e-konsultasjon til fastlegen?

a. Fastlegen eller legesekretær
b. Brosjyrer, reklame eller skriftlig informasjon om e-konsultasjoner
c. Venner, familie eller kollegaer
d. Funnet ut av det selv, for eksempel pa Helsenorge eller fastlegekontorets hjemmeside
e. Annet

5) Hva handlet e-konsultasjonen om? (flere svar er mulig)

a. Jeg spurte om jeg trenger å bestille en legetime
b. Spørsmål om nylig oppstått helseproblem
c. Spørsmål om helseproblem som jeg tidligere har diskutert med fastlegen min
d. Medisinbruken min
e. Sykmelding eller en attest
f. Prøvesvar eller en undersøkelse, f.eks røntgen
g. Svarte på en melding fra fastlegen min
h. Annet

6) Hvor bekymret er du for problemet du sendte e-konsultasjon om?

a. Ikke bekymret
b. Noe bekymret
c. Veldig bekymret

7) Dersom du ikke hadde hatt muligheten til a sende en e-konsultasjon til fastlegen, hva ville du gjort?

a. Bestilt en legetime
b. Ringt legekontoret
c. Kontaktet legevakten
d. Søkt informasjon på internett eller andre steder om problemet mitt
e. Ventet litt
f. Annet

8) Tror du fastlegen vil svare på denne e-konsultasjonen ved å be deg komme inn på legekontoret for en undersøkelse?

a. Ja
b. Nei
c. Vet ikke

9) Når forventer du å få svar på denne e-konsultasjonen?

a. Innen 12 timer
b. Innen 24 timer
c. Innen 48 timer
d. Mellom 48 timer og fem døgn

10) Hvor enig eller uenig er du i følgende påstander?
(Helt enig, enig, verken enig eller uenig, uenig, helt uenig)

a. Å sende denne e-konsultasjonen var mitt førstevalg for å få svar fra fastlegen om mitt problem fastlegen om mitt problem
b. Det er vanligvis lett å komme gjennom på telefon til mitt legekontor
c. Jeg får vanligvis legetime innen rimelig tid

11) Alt i alt, hvor fornøyd var du med til å kontakte fastlege med en e-konsultasjon i dag?

a. Veldig fornøyd
b. Fornøyd
c. Verken fornøyd eller misfornøyd
d. Misfornøyd
e. Veldig misfornøyd

12) Har du forslag til noe som kan gjøre tjenesten e-konsultasjon med fastlegen bedre? (fritekst)

13) Hvor mange e-konsultasjoner har du sendt til fastlegen de siste 12 manedene, inkludert denne?

g. 1-3
h. 4-9
i. 10-19
j. 20 eller flere

14) Hvis svar: a. 1-3 på spørsmål 13: Er dette din første e-konsultasjon, noensinne?

a. Ja
b. Nei

15) Hvor mange legetimer har du hatt på fastlegekontoret de siste 12 månedene?

a. 0-3
b. 4-9
c. 10-19
d. 20 eller flere

16) Hvor lang tid tar det deg vanligvis a dra til legen (reisetid fra d0r til d0r)?

a. 0-30 minutter
b. 30-60 minutter
c. 1-2 timer
d, Mer enn 2 timer

Til slutt noen bakgrunnsspørsmål om deg:

17) Hvilket kjønn er du?

a. Kvinne
b. Mann
c. Annet/ønsker ikke å svare

18) Hvor gammel er du?

a. 16-25 år
b.26-40 år
c. 41-55 år
d. 56-70 år
e. 71-85 år
f. Over 85 år

19) Hva er din høyest utførte utdanning?

a. Grunnskole 10 år eller mindre (barne- og ungdomsskole, framhaldskole)
b. Videregående skole (realskole, middelskole, yrkesskole)
c. Fagskole/svennebrev
d. Universitetsutdanning mindre enn 4 år
e. Universitetsutdannelse 4 år eller mer
f. Annet
