# Supplementary material for: Patients’ Use of e-Consultations as an Alternative to Other General Practitioner Services: Cross-Sectional Survey Study
Source: J Med Internet Res. 2025 Jan 8;27:e55158. doi: 10.2196/55158 (PMC11754976; doi:10.2196/55158)
Supplement: Multimedia Appendix 4 [file jmir_v27i1e55158_app4.pdf]

Cross-tables divided by alternative action and patient/e-consultation characteristics. N=13011

	Book GP appointment n=5917 (45.5)	Call GP front desk n=5846 (44.9)	Out-of-hours service n=323 (2.5)	Wait or seek information n= 609 (4.7)	Other n= 316 (2.4)	Total n=13011 (100)
	N (%)	n (%)	n (%)	n (%)	n (%)	n (%)
**Background patient characteristics**						
Gender						
Female	4045 (68.4)	4182 (71.5)	202(62.5)	420 (69)	210 (66.5)	9059 69.6)
Male	1831 (30.9)	1625 (27.8)	116 (35.9)	178 (26.2)	98 (31)	3848 (29.6)
Other/no answer	41 (0.7)	39 (0.6)	5 (2.6)	11 (4.8)	8(2.5)	104 (0.8)
Age						
16-25 years	296 (5)	402 (6.9)	28 (8.7)	65 (10.7)	32 (10.1)	823 (6.3)
26-40 years	1564 (26.4)	1654 (28.3)	95 (29.4)	211 (34.6)	71 (22.5)	3595 (27.6)
41-55 years	2277 (38.5)	2092 (35.8)	109 (33.7)	175 (28.7)	111 (35.2)	4764 (36.6)
56-70 years	1465 (24.8)	1396 (23.9)	72 (22.3)	113 (18.6)	77 (24.4)	3123 (24)
Over 70 years	315 (5.3)	302 (5.2)	19 (5.9)	45 (7.4)	25 (7.9)	706 (5.4)
Highest completed education level						
Non-university	2496 (42,2)	2665 (45,6)	163 (50,5)	253 (41,5)	141 (44,6)	5718 (43.9)
University	3320 (56,1)	3069 (52,5)	152 (47,1)	332 (54,5)	158 (50)	7031 (54)
Other	101 (1,7)	112 (1,9)	8 (2,5)	24 (2,9)	17 (5,4)	262 (2)
Appointments at the GP office last 12 months						
0-3	2869 (48,5)	2758 (47,2)	148 (45,8)	303 (49,8)	158 (50)	6236 (47,9)
4 -9	2529 (42,7)	2545 (43,5)	134 (41,5)	244 (40,1)	120 (38)	5572 (42,8)
10-19	451 (7,6)	463 (7,9)	30 (9,3)	52 (8,5)	33 (10,4)	1029 (7,9)
20 or more	68 (1,1)	80 (1,4)	11 (3,4)	19 (1,6)	5 (1,6)	17 4(1,3)
E-consultations last 12 months, including this						
1-3	3059 (51.7)	2928 (50.1)	167 (51.7)	314 (51.6)	178 (56.3)	6646 (51.1)
4-9	2139 (36.2)	2071 (35.4)	107 (33.1)	204 (33.5)	86 (27.4)	4607 (35.4)
10-19	565 (9.5)	657 (11.29	38 (11.8)	66 (10.8)	32 (10.1)	1258 (10.4)
20 or more	154 (2.6)	190 (3.3)	11 (3.4)	25 (4.1)	20 (6.3)	400 (3.1)
**Availability of GP services**						
Travel time to GP office						
0-30 min	4652 (78.6)	4606 (78.8)	207 (64.1)	455 (74.7)	235 (74.4)	10155 (78)
30-60 min	993 (16.8)	950 (16.3)	75 (23.2)	106 (17.4)	54 (17.1)	2178 (16.7)
1-2 hours	201 (3.4)	201 (3.4)	22 (6.8)	30 (4.9)	14 (4.4)	468 (3.6)
>2 hours	71 (1.2)	89 (1.5)	19 (5.9)	18 (3)	13 (4.1)	210 (1.6)
I usually get through on the phone to my doctor's front desk						
Agree	2780 (47)	2813 (48.1)	119 (36.8)	251 (41.2)	132 (41.8)	6095 (46.8)
Neither nor	1532 (25.9)	1464 (25)	87 (26.9)	185 (30.4)	97 (30.7)	3365 (25.9)
Disagree	1605 (27.1)	1559 (26.8)	117 (36.2)	173 (28.4)	87 (27.5)	3551(27.3)
I usually get a GP appointment within a reasonable time						
Agree	3819 (64.5)	3841 (65.7)	152 (47.1)	380 (62.4)	174 55.1)	8366 (64.3)
Neither nor	1132 (19.1)	1095 (18.7)	93 (28.8)	122 (20)	71 (22.5)	2513 (19.3)
Disagree	966 (16.3)	910 (15.6)	78 (24.1)	107 (17.6)	71 (22.5)	2132 (16.4)
**Use and access to e-consultations**						
Who told you it is possible to send an e-consultation to the GP?						
Figured it myself	3357 (56.7)	3355 (57.4)	177 (54.8)	403 (66.2)	1 74 (55.1)	7479 (57.4)
GP/receptionist	2129 (36)	2042 (34.9)	120 (37.2)	150 (24.6)	112 (35.4)	

Written info/ad	47 (0.8)	47 (0.8)	2 (0.6)	5 (0.8)	0 (0)	4553 (35.0)
Friends/ family	275 (4.6)	308 (5.3)	16 (5)	38 (6.2)	15 (4.7)	101 (0.8)
Other	109 (1.8)	94 (1.6)	8 (2.5)	13 (2.1)	15 (4.7)	652 (5.0)
						239 (1.8)
Sending a e-consultation was my first choice to get an answer from my GP about my problem today						
Agree	4820 (81.5)	4614 (78.9)	234 (72.4)	510 (83.7)	191 (60.4)	10369 (79.7)
Neither nor	615 (10.4)	642 (11)	38 (11.8)	60 (9.9)	55 (17.4)	1410 (10.8)
Disagree	482 (8.1)	590 (10.1)	51 (15.8)	39 (6.4)	70 (22.2)	1232 (9.5)
Expected time to get an answer to this e-consultation						
Within 12 hours	1778 (30)	2234 (38.2)	154 (47.7)	178 (29.2)	102 (32.2)	4445 (34.2)
Within 24 hours	2020 (34.1)	1979 (33.9)	103 (31.9)	194 (31.9)	69 (21.8)	4365 (33.5)
Within 48 hours	1211 (20.5)	927 (15.9)	29 (9)	110 (18.1)	63 (19.9)	2340 (18)
Between 48 hours and 5 days	908 (15.3)	706 (12.1)	37 (11.5)	127 (20.9)	82 (25.9)	1860 (14.3)
Getting the e-consultation for free?						
Yes	511 (8.6)	552 (8.9)	24 (7.4)	51 (8.4)	32 (10.1)	1140 (8.8)
No	4389 (74.2)	3992 (68.3)	225 (69.7)	413 (678)	182 (57.6)	9201 (70.7)
Don't know	1917 (17.2)	1332 (22.8)	74 (22.9)	145 (23.8)	102 (32.2)	2670 (20.5)
To the ones that got the e-consultation for free: Would you sent if you had to pay?						
Yes	413 (80.8)	384 (73.6)	18 (75)	30 (58.8)	25 (78.1)	870 (76.3)
No	59 (9.8)	75 (14.4)	3 (12.5)	10 (19.6)	3 (9.4)	141 (12.4)
Don't know	48 (9.4)	63 (12.1)	3 (12.5)	11 (21.6)	4 (12.5)	129 (11.3)
**Characteristics of the problem handled in the e-consultation.**						
How concerned are you about the issue you sent an e-consultation about?						
Not worried	1927 (32.6)	2410 (41.2)	57 (17.6)	226 (37.1)	124 (39.2)	4744 (36.5)
Somewhat worried	3152 (53.3)	2714 (46.4)	150 (46.4)	313 (51.4)	114 (36.1)	6443 (49.5)
Very worried	838 (14.2)	722(12.4)	116 (35.9)	70 11.5)	78 (24.7)	1824 (14)
Do you think the GP answer this e-consultation by asking you to come into the office for a physical examination?						
Yes	1102 (18.6)	969 (16.6)	84 (26)	99 (16.3)	56 (17.7)	2310 (17.8)
No	2261 (38.2)	2694 (46.1)	67 (20.7)	232 (38.1)	142 (44.9)	5396 (41.5)
Don't know	2554 (43.2)	2183 (37.3)	172 (53.3)	278 (45.6)	118 (37.3)	5305 (40.8)
What was the e-consultation about?						
Sick certificates	2233 (37.7)	2448 (41.9)	99 (30.7)	97 (15.9)	63 (19.9)	4940 (38)
Known problem	2578 (43.6)	1809 (30.9)	104 (32.2)	207 (34)	80 (25.3)	4778 (36.7)
New problem	1313 (22.2)	1254 (21.5)	157 (48.6)	234 (38.4)	57 (18)	3015 (23.2)
Medication use	728 (12.3)	704 (12)	36 (11.1)	97 (15.9)	30 (9.5)	1595 (12.3)
Other	596 (10.1)	705 (12.1)	25 (7.7)	85 (14)	126 (39.9)	1537 (11.8)
Asking triage	521 (8.8)	482 (8.2)	40 (12.4)	56 (9.2)	11 (3.5)	1110 (8.5)
Test results	392 (6.6)	467 (8)	12 (3.7)	53 (8.7)	19 (6)	943 (7.2)
Answered e-con	186 (3.1)	210 (3.6)	12 (3.7)	29 (4.8)	16 (5.1)	453 (3.5)
Sending e-consultation on behalf of…						
Themselves	5437 (91.9)	5249 (89.9)	283 (87.6)	563 (92.4)	296 (93.7)	11828 (90.9)
Their child	440 (7.4)	539 (9.2)	34 (10.5)	44 (7.2)	11 (3.5)	1968 (8.2)
Others you have power of attorney for	40 (0.7)	58 (1)	6 (1.9)	2 (0.3)	9 (2.8)	11 5(0.9)
All in all. how satisfied were you with contacting the GP through an e-consultation today?						

Satisfied	5136 (86.8)	4848 (82.9)	223 (69)	507 (83.3)	199 (63)	10913 (83.9)
Neither nor	680 (11.5)	846 (14.5)	87 (26.9)	86 (14.1)	77 (24.4)	1776 (13.6)
Dissatisfied	101 (1.7)	152 (2.6)	13 (4)	16 (2.6)	40 (12.7)	322 (2.5)
